# Comprehensive Germline Profiling of High-Grade Serous Ovarian Cancer Using Whole-Exome Sequencing

**DOI:** 10.3390/ijms27125564

**Published:** 2026-06-19

**Authors:** Hye-Lim Cho, Seong Eun Bak, Mi-Ryung Han, Youn Jin Choi

**Affiliations:** 1 Division of Life Sciences, College of Life Sciences and Bioengineering, Incheon National University, Incheon 22014, Republic of Korea; joao216@inu.ac.kr; 2 Department of Obstetrics and Gynecology, Bucheon St. Mary’s Hospital, College of Medicine, The Catholic University of Korea, Seoul 06591, Republic of Korea; newsseong@gmail.com; 3 Institute for New Drug Development, College of Life Science and Bioengineering, Incheon National University, Incheon 22014, Republic of Korea; 4 Department of Obstetrics and Gynecology, Seoul St. Mary’s Hospital, College of Medicine, The Catholic University of Korea, Seoul 06591, Republic of Korea; 5 Cancer Research Institute, College of Medicine, The Catholic University of Korea, Seoul 06591, Republic of Korea

**Keywords:** germline variants, somatic variants, high-grade serous ovarian cancer, hereditary breast and ovarian cancer, family history, screening, whole-exome sequencing, clonal hematopoiesis, propensity score matching

## Abstract

While ovarian cancer screening is not recommended in the general population, attention has shifted to screening women with elevated hereditary risks. Although germline *BRCA 1/2* pathogenic variants account for 40% of inherited ovarian cancer risk and family history (FH) remains important, known germline variants alone do not fully explain familial ovarian cancer risk. Whole-exome sequencing (WES) was performed on blood samples taken from 231 individuals, including 39 patients with high-grade serous ovarian cancer (HGSOC) and 192 healthy controls (HCs) stratified by FH. We analyzed pathogenic or likely pathogenic (P/LP) germline variants in cancer-related genes and assessed their association with family cancer history. Additionally, we performed somatic variant comparisons using 1:4 propensity score matching and analyzed clonal hematopoiesis of indeterminate potential (CHIP)-related somatic variants. P/LP germline variants were detected in 56.4% of HGSOC patients, 49.4% of controls with FH, and 33.3% without. The HGSOC group and controls with FH exhibited similar P/LP germline mutation patterns in ovarian cancer-related genes. From CHIP analysis, somatic CHIP mutations were detected in 6.3% of the HGSOC group and 8.5% in HCs. Our findings demonstrate genomic overlap between ovarian cancer patients and FH-positive individuals. Therefore, germline variant screening could be considered to facilitate early diagnosis.

## 1. Introduction

Globally, ovarian cancer is the third most common gynecological malignancy and the second leading cause of death in this category [1]. Due to early detection challenges, approximately 80% of cases are diagnosed at stages III or IV, resulting in a 5-year survival rate of less than 45% [2]. Large-scale ovarian cancer screening studies in the United Kingdom (UK) (e.g., the UK Collaborated Trial of Ovarian Cancer Screening trial) have examined transvaginal sonography and tumor markers (e.g., CA125) in the general population to enhance survival outcomes. This long-term follow-up study reported limited effectiveness and concluded that routine screening was not warranted in the general population [3]. Thus, screening efforts have shifted toward women with elevated hereditary risk, as reflected in National Comprehensive Cancer Network (NCCN) guidelines for Genetic/Familial High-Risk Assessment: Breast, Ovarian, Pancreatic, and Prostate [4]. Family history (FH) remains an important inherited susceptibility marker [5], although rare pathogenic variants in high- and moderate-penetrance susceptibility genes, including *BRCA 1/2*, and related genes, are estimated to account for approximately 40% of the inherited component in epithelial ovarian cancer risk. In contrast, common susceptibility variants identified in genome-wide association studies contribute a smaller proportion of heritability, estimated at around 6%. Notably, germline mismatch repair gene variants are more strongly associated with specific histological subtypes, particularly endometrioid and clear cell ovarian cancers [6].

Recent multi-gene panel testing trends show that *BRCA 1/2* mutations, while most frequent, account for only a small portion of germline pathological variants (PVs), and homologous recombination-related genes (e.g., *RAD51C, RAD51D, BRIP1, PALB2*, and *ATM*) collectively contribute a substantial proportion to mutation-positive cases [7]. Germline PVs exhibit distinct clinical–pathological associations, with homologous recombination repair-related PVs more strongly linked to serous histology and advanced stages, and also associated with improved overall survival in stage III/IV disease, supporting a need for variant-specific risk stratification and treatment strategies [8]. This suggests that known pathogenic and likely pathogenic (P/LP) germline variants alone do not fully explain familial ovarian cancer risk, and requires the identification of additional contributing factors [9].

Family history-positive but unaffected individuals may represent a genetically enriched population with inherited susceptibility architectures that overlap with those observed in cancer patients, despite the absence of clinically manifest disease. Because hereditary cancer predisposition demonstrates incomplete penetrance and age-dependent expression, unaffected individuals with a family history of hereditary breast and ovarian cancer(HBOC)-related cancers may still harbor pathogenic germline variants and related genomic instability profiles. Therefore, evaluating genomic similarities between ovarian cancer patients and healthy individuals with a family history may provide important insights into inherited cancer susceptibility beyond currently established high-penetrance genes [9,10,11].

This study investigated the extent to which P/LP variants could explain familial ovarian cancer risk and evaluated whether FH remains an independent risk factor beyond known genetic variants.

## 2. Results

### 2.1. Study Population

In total, 39 patients with HGSOC and 192 healthy controls (HCs) were included, comprising 156 females in the HCs group with a FH of cancer (HC-FH(+)) and 36 HCs without a family history (HC-FH(−)). HC-FH(+) refers to HCs with a FH of breast, ovarian, prostate, or pancreatic cancer in third-degree relatives, whereas HC-FH(−) refers to those without FH. FH was assessed up to third-degree relatives in accordance with NCCN guidelines for hereditary breast and ovarian cancer risk assessment [4]. Clinical information is shown (Table 1). The mean age was 57.41 ± 11.65 years for the HGSOC group, 39.9 ± 11.51 years for the HC-FH(+) group, and 51.17 ± 9.71 years for the HC-FH(−) group. Age distribution varied across groups, with most (80.2%) HC-FH(+) participants being younger (predominantly in the 30–39 age range), whereas HGSOC and HC-FH(−) participants were more frequently in the 40–69 age range (88.9%). The underlying disease prevalence for conditions such as hypertension, diabetes, thyroid disease, and rheumatic disease was 30.7% in the HGSOC group, 27.5% in the HC-FH(+) group, and 13.9% in the HC-FH(−) group. In patients with HGSOC, three individuals (7.7%) were concurrently diagnosed with HGSOC and either breast (*n* = 2) or thyroid cancer (*n* = 1). In the HC-FH(+) group, 70.5% had a FH of breast cancer, 40.4% a FH of ovarian cancer, 1.3% a FH of prostate cancer, and 3.8% a FH of pancreatic cancer in third-degree relatives.

### 2.2. The Germline Mutation Landscape in the Study Population

**Figure 1:**
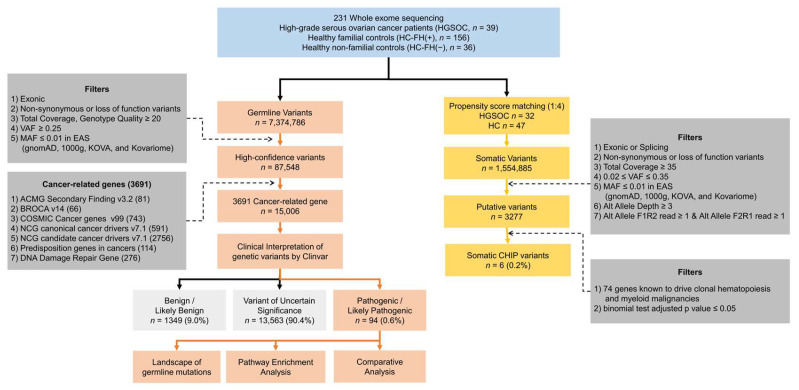
The workflow. We included 231 participants. “Healthy familial controls (HC-FH(+))” refers to samples from healthy individuals with a family history (FH) of breast cancer (BC), ovarian cancer (OV), prostate cancer (PC), or pancreatic cancer (PA) in third-degree relatives, whereas “healthy non-familial controls (HC-FH(−))” refers to samples from healthy individuals with no known FH of these cancers. Germline and somatic variants analyses are described (Supplementary Method, available online). The solid arrows represent the workflow, and the dashed arrows represent the variant filters. Abbreviations: HGSOC, High-grade serous ovarian cancer; HC, Healthy control; FH, Family history; VAF, Variant allele frequency; MAF, Minor allele frequency; gnomAD, Genome Aggregation Database; 1KGP, 1000 Genomes Project; KOVA, Korean Variant Archive; Kovariome, Korean National Standard Reference Variome; EAS, East Asian; ACMG, American College of Medical Genetics and Genomics; SF, Secondary findings; COSMIC, Catalog Of Somatic Mutations In Cancer; NCG, Network of Cancer Genes; CHIP, clonal hematopoiesis of indeterminate potential.

Samples (*n* = 231) were analyzed using whole-exome sequencing (WES), with a mean sequencing depth of 112× (range = 80–188×). The study workflow is shown (Figure 1). In total, 7,374,786 germline variants were identified (mean/sample = 31,926; range = 31,294–32,481). After applying filtering criteria for high-quality and rare coding variants using public population databases, 87,548 high-confidence germline variants remained. Through systematic investigations, we selected 3691 cancer-related genes based on the following criteria: (1) Cancer gene databases including the Catalog of Somatic Mutations in the Cancer Gene Census (COSMIC) and Network of Cancer Genes (NCG); (2) Genes associated with cancer predisposition; (3) Actionable genes recommended for reporting by the American College of Medical Genetics and Genomics (ACMG); and (4) DNA repair genes [12,13,14,15,16,17]. Of the 87,548 high-confidence germline variants, 15,006 unique variants were mapped to 3066/3691 cancer-related genes, and classified into three categories based on the ClinVar database: P/LP (*n* = 94, 0.6%), variant of uncertain significance (VUS) (*n* = 13,563, 90.4%), and benign or likely benign (*n* = 1349, 9.0%). Among these, 94 P/LP germline variants were retained for further analyses (Table S1).

### 2.3. Associations Between P/LP Germline Mutations and a Cancer FH

We analyzed HGSOC, HC-FH(+), and HC-FH(−) groups to identify differences in P/LP germline mutation in cancer-related genes.

**Figure 2:**
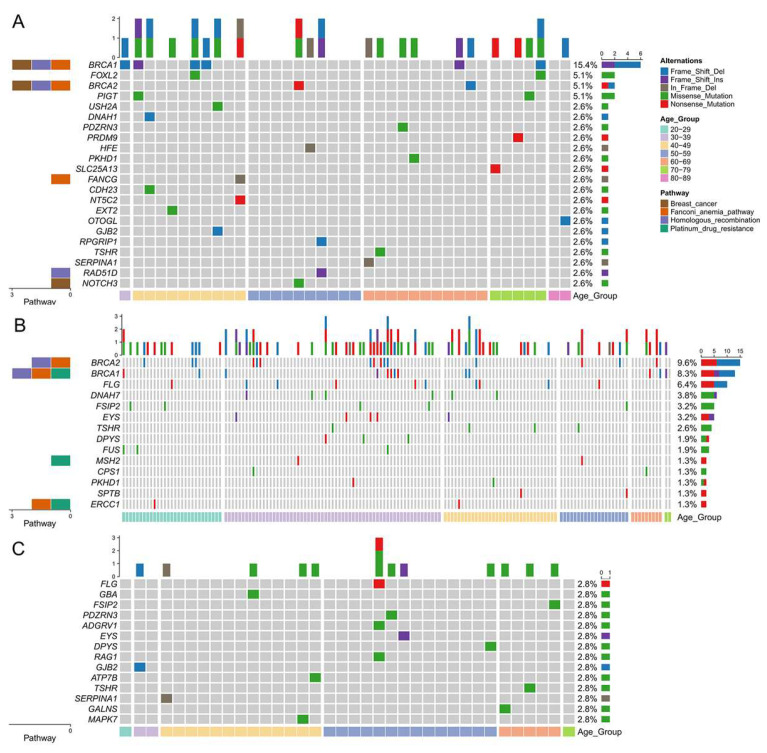
The P/LP germline variants landscapes 231 participants. Row represents genes from cancer-related gene lists, and each column corresponds to a participant. The upper plot shows the number of P/LP germline variants in participants. The bar plot (**right**) shows the number of P/LP germline variants in each gene and the percentages represent the proportion of participants with a mutation in each gene. The bottom section displays the subject’s age status. The left-side plot displays the number of pathways associated with genes using pathway enrichment analysis of P/LP germline mutations in each group. Genes with frequencies exceeding 1% in groups are shown. (**A**) For the HGSOC group, 27 P/LP germline variants in 22 genes were observed. (**B**) For the HC-FH(+) group, 69 P/LP germline variants in 41 genes were identified. (**C**) For the HC-FH(−) group, 14 P/LP germline variants in 14 genes were identified. Abbreviations: P/LP, Pathogenic/Likely pathogenic; HC, Healthy control; Del, Deletion; Ins, Insertion.

Among 39 patients with HGSOC, 22 (56.4%) carried 27 P/LP variants in 22 cancer-related genes (Figure 2 and Figure S1, Table S1). The most frequently mutated genes (≥4% of samples) were *BRCA1* (15.4%), *FOXL2* (5.1%), *BRCA2* (5.1%), and *PIGT* (5.1%) (Figure 2). Kyoto Encyclopedia of Genes and Genomes (KEGG) analysis identified three significant pathways involving these 22 genes: the breast cancer pathway (*BRCA1*, *BRCA2*, and *NOTCH3*), the Fanconi anemia pathway (*BRCA1*, *BRCA2*, and *FANCG*), and the homologous recombination pathway (*BRCA1*, *BRCA2*, and *RAD51D*).

Among the 156 HCs with a FH (HC-FH(+)), 77 (49.4%) carried 69 P/LP variants in 41 cancer-related genes (Figure 2 and Figure S2, Table S1). In this group, *BRCA2* (9.6%), *BRCA1* (8.3%), and *FLG* (6.4%) were the most frequently mutated genes (≥ 4% of samples) (Figure 2). KEGG analysis of the 41 genes identified three significant pathways similar to the HGSOC cohort: the Fanconi anemia pathway (*BRCA1*, *BRCA2*, *ERCC1*, *POLH*, and *BRIP1*), the homologous recombination pathway (*BRCA1*, *BRCA2*, *ATM*, and *BRIP1*), and the platinum drug resistance pathway (*BRCA1*, *MSH2*, *ERCC1*, *POLH*, *ATM*, and *ATP7B*). Among the 36 HCs without a FH (HC-FH(−)), 12 (33.3%) carried 14 P/LP variants in 14 cancer-related genes; however, no significantly enriched pathways were identified (Figure 2 and Figure S3, Table S1).

Taken together, the HGSOC and HC-FH(+) groups exhibited similar P/LP germline mutation patterns, particularly in ovarian cancer-related genes or pathways, which differed from the HC-FH(−) group. These findings support the well-established notion that FH is a significant factor associated with P/LP germline mutation status in ovarian cancer [10,11,18].

### 2.4. Comparative Analysis of Germline Mutations Using Public Genomic Databases

We independently performed a comparative analysis of germline mutations using several public genomic databases. Specifically, we examined which P/LP germline mutations in cancer-related genes occurred at significantly higher frequencies in groups when compared to the control group. The HGSOC, HC-FH(+), and HC-FH(−) groups were used as case groups, while the public gnomAD database served as the control group for each comparison. After adjusting *p*-values in case groups, 50/94 P/LP germline mutations occurred at significantly higher frequencies in at least one case group when compared to the control group (Table S2). Among the 50 P/LP germline mutations, 12 (12/50, 24%) were detected in the HGSOC group, 40 (40/50, 80%) in the HC-FH(+) group, and four (4/50, 8%) in the HC-FH(−) group.

In the HGSOC group, 12 P/LP mutations were identified in seven cancer-related genes, with the most frequently mutated genes being *BRCA1* (5/12, 41.7%) and *BRCA2* (2/12, 16.7%). Additional mutations were observed in *PIGT*, *USH2A*, *NT5C2*, *PRDM9*, and *SLC25A13* (1/12, 8.3% each). In the HC-FH(+) group, 40 P/LP mutations were detected in 22 cancer-related genes. The most recurrently mutated gene was *BRCA2* (10/40, 25.0%), followed by *BRCA1* (8/40, 20.0%) and *ERCC1* (2/40, 5.0%). Other mutations were identified in *ADAMTS17*, *ATM*, *COL7A1*, *DYSF*, *FBN1*, *FLG*, *FUS*, *MSH2*, *NTHL1*, *TRIM37*, *CENPJ*, *NHEJ1*, *TSHR*, *VARS2*, *DNAH7*, *CPS1*, *EYS*, *ATP8B1*, and *PKHD1* (1/40, 2.5% each). In the HC-FH(−) group, four P/LP germline mutations were detected in four cancer-related genes: *GBA*, *ADGRV1*, *GALNS*, and *FLG* (1/4, 25.0% each).

**Figure 3:**
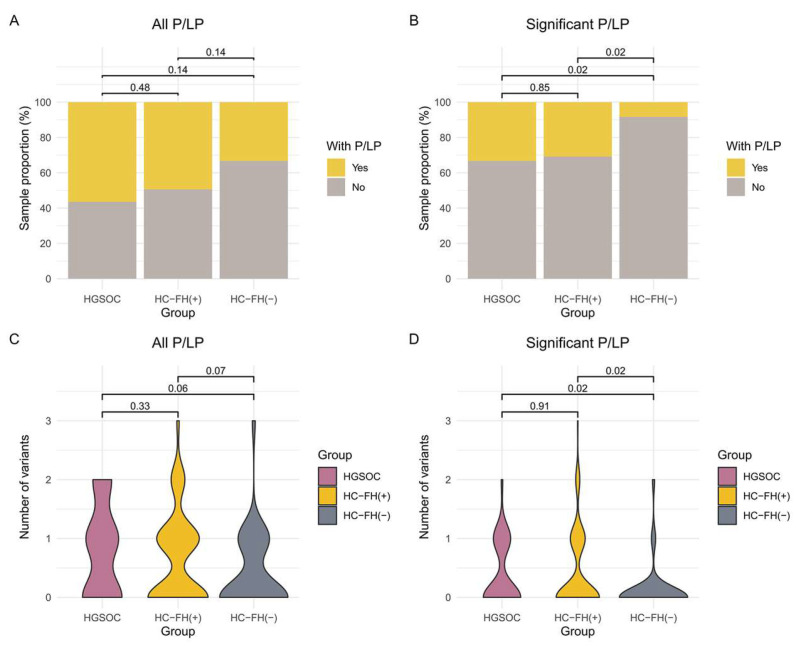
P/LP germline mutation profiles across HGSOC, HC-FH(+), or HC-FH(−) groups. Within the entire cohort, ‘All P/LP’ refers to a total of 94 germline variants (listed in Table S1), while ‘Significant P/LP’ indicates a subset of 50 variants filtered through comparative analysis (listed in Table S2). The barplot shows the proportion of samples harboring cancer-related P/LP germline mutations in HGSOC, HC-FH(+), and HC-FH(−) groups. The violin plot represents the distribution of the number of cancer-related P/LP germline mutations per sample in HGSOC, HC-FH(+), and HC-FH(−) groups. The *p*-value was estimated using a pairwise two-sided Fisher exact test or Wilcox test. For multiple testing correction, a Benjamini–Hochberg adjusted *p*-value < 0.05 was considered significant. (**A**) Sample proportion in groups carrying any cancer-related P/LP germline mutations. (**B**) Sample proportion in groups carrying significant cancer-related P/LP germline mutations in comparative analysis *. (**C**) Distribution of the number of any cancer-related P/LP germline mutations per sample across the three groups. (**D**) Distribution of the number of significant cancer-related P/LP germline mutations per sample across the three groups using comparative analysis *. * In comparative analysis, we examined which P/LP germline mutations in cancer-related genes showed significantly higher frequencies in groups when compared to the public control group (gnomAD). Abbreviation: HGSOC, High-grade serous ovarian cancer; HC, Healthy control; FH, Family history; P/LP, Pathogenic/Likely pathogenic; HC, Healthy control; gnomAD, Genome Aggregation Database.

We next conducted a pairwise comparative analysis of P/LP germline mutation profiles across the three groups. No significant associations were found between P/LP germline mutations in cancer-related genes and any specific group (Figure 3A, Table S3). Additionally, no significant differences were observed in the median number of P/LP germline variants per sample across groups (Figure 3C). However, in comparative analyses, a P/LP germline mutation was significantly associated with both HGSOC (odds ratio [OR]: 5.38, 95% confidence interval [CI]: 1.29–32.50, adjusted *p*-value < 0.05) and HC-FH(+) (OR: 4.86, 95% CI: 1.42–26.00, adjusted *p*-value < 0.05) groups when compared to the HC-FH(−) group (Figure 3B, Table S3). Furthermore, the median number of P/LP germline variants identified in comparative analyses was significantly higher in the HGSOC and HC-FH(+) groups when compared to the HC-FH(−) group (adjusted *p*-value < 0.05, Figure 3D). Therefore, the HGSOC group exhibited a P/LP germline mutation profile more similar to the HC-FH(+) group than to the HC-FH(−) group, in terms of both mutated genes and mutation frequencies.

### 2.5. Clonal Hematopoiesis Analysis

To further investigate potential genomic differences between HGSOC and HC groups, we performed an exploratory clonal hematopoiesis of indeterminate potential (CHIP) analysis. To account for the impact of age and FH, 32 patients with HGSOC were matched to 47 HCs using 1:4 propensity score matching (PSM). After this, mean ages in HGSOC and HC groups were 53.9 ± 9.1 years and 52.5 ± 8.7 years, respectively (Table S4).

In the matched cohort, we identified 1,554,885 somatic variants using WES, with a mean of 19,683 variants per sample (range = 8842–184,396). After applying filtering criteria for high-quality and rare coding variants based on public population databases, 3277 putative somatic variants were retained. From this set, six variants were selected based on a predefined variant list of 74 genes (Table S5) known to drive clonal hematopoiesis and myeloid malignancies [19]. Next, a binomial model was applied to each putative somatic CHIP mutation to assess whether the variant allele frequency (VAF) significantly differed from the germline mutation distributions. All six putative CHIP mutations had adjusted *p*-values < 0.05 and were classified as somatic.

Of the 79 participants (32 patients with HGSOC, 47 HCs) in this analysis, six (7.6%) harbored one somatic CHIP mutation; two in the HGSOC group (2/32, 6.3%), and four in the HC group (4/47, 8.5%). The mean VAF for CHIP was 3.6% (range = 2.5–4.7%) in the HGSOC group, and 6.9% (range = 3.1–12.7%) in the HC group. Among the six CHIP carriers, the most frequently mutated gene was *DNMT3A* (2/6, 33.3%), followed by *TET2, STAG2, SMC3*, and *CREBBP* (1/6, 16.7% each) (Figure S4). In the two patients carrying CHIP mutations in the HGSOC group, two (100%) carried three P/LP germline mutations in cancer-related genes, including *BRCA1*, *FANCG*, and *NT5C2*. In the HC group, four individuals harbored CHIP mutations, but only one (25%) had a P/LP germline mutation in a cancer-related gene, *EYS*.

We next performed a multivariate logistic regression analysis controlling for age and FH in the matched cohort to evaluate the independent cancer risk of CHIP and germline variants. To minimize subjective interpretation relating to germline variants, we selected P/LP germline variants in ovarian cancer-related genes recommended by NCCN guidelines [4]. Notably, ovarian cancer-related P/LP germline variants were significantly associated with increased cancer risk (OR = 15.53, CI = 2.90–176.53, *p*-value < 0.01), whereas CHIP status did not exhibit any statistically significant association (OR = 0.55, CI = 0.06–3.37, *p*-value = 0.55) (Table S6). Taken together, P/LP germline variants in ovarian cancer-related genes appear to have critical roles in developing cancer rather than CHIP, thereby emphasizing the importance of genetic screening regardless of age and FH.

## 3. Discussion

We highlight the importance of germline variants in ovarian cancer susceptibility. By leveraging WES in a well-characterized cohort, we investigated associations between P/LP germline variants and a FH of HBOC-related malignancies. Our analysis revealed an overlap in germline variant profiles between ovarian cancer patients and HCs with a FH of HBOC-related malignancies. These findings enhance our understanding of the genetic risk landscape in ovarian cancer and identify candidate genes that require further functional studies.

Approximately 10% or more of patients diagnosed with high-grade serous epithelial ovarian cancer harbor germline or somatic mutations in *BRCA1* or *BRCA2* [20]. In our study, we observed that 20.5% of patients with HGSOC (8/39) had P/LP germline *BRCA1* or *BRCA2* mutations. Similarly, 17.3% of HCs with a FH of HBOC-related cancers (27/156) harbored P/LP germline *BRCA1* or *BRCA2* mutations. We identified five P/LP germline *BRCA1/BRCA2* mutations and three P/LP germline variants in *PKHD1*, *SLC25A13*, and *OTOGL* in both groups. *PKHD1* alterations have been reported in genomic sequencing studies investigating rare ovarian malignancies, where somatic or germline variants may act as cooperative factors in tumorigenesis [21]. *SLC25A13* encodes a mitochondrial solute carrier and is linked to metabolic reprogramming in cancers, potentially contributing to tumor cell survival [22,23]. Since *BRCA1/2* mutations do not always appear in patients with HGSOC, identifying and characterizing novel screening targets could advance precision oncology for the broader ovarian cancer spectrum.

Pathway enrichment analyses on the mutated genes revealed prominent clustering in DNA damage response and oncogenic signaling networks. Notably, among the three significantly enriched pathways in HGSOC, homologous recombination and Fanconi anemia pathway were overrepresented in the HC-FH(+) group. HGSOC is a malignancy characterized by chromosomal instability that arises from a deficient homologous recombination repair pathway [24]. The Fanconi anemia pathway is crucial for repairing DNA interstrand crosslinks and mediating cellular adaptations to replication stress [25]. These pathway enrichment profiles fundamentally underscore the critical role of genomic instability in driving ovarian carcinogenesis, while clinically demonstrating that individuals with a FH harbor significantly elevated risks of developing genomic fragility.

Comparative analysis using the gnomAD database revealed that 50 P/LP germline variants were significantly enriched in at least one of our groups relative to the general population. Of the 50 P/LP germline variants, five (three in *BRCA1* and two in *BRCA2*) were significantly more prevalent in HGSOC and HC-FH(+) groups when compared to the gnomAD database. Furthermore, when evaluating these 50 variants across all three groups, a comprehensive analysis of the proportion of mutation-positive individuals and the cumulative number of variants per sample revealed distinct stratification. This genomic HC-FH(+) burden was statistically similar to that in patients with HGSOC, but both groups showed a significantly higher genomic burden when compared to the HC-FH(−) group. By investigating specific germline variants that were significantly enriched in groups, our results suggest key genetic susceptibility factors contributing to an elevated risk of ovarian tumorigenesis.

As an exploratory component of this study, we attempted to profile CHIP in our cohorts to evaluate its impact on ovarian carcinogenesis. CHIP is fundamentally characterized as an age-dependent phenomenon [26]. Thus, we examined CHIP in a matched dataset by carefully controlling for age and FH. In our dataset, which was matched at a mean age of approximately 53 years, we identified a CHIP prevalence of 6.3% (2/32) in the HGSOC cohort and 7.6% (4/47) in the HC group. Two HGSOC patients with CHIP (diagnosed at ages 34 and 43) carried P/LP germline variants involved in homologous recombination and Fanconi anemia pathway, including *BRCA1* and *FANCG*. This co-occurrence possibly indicated a potential association between CHIP and tumor progression, although its clinical significance remains unclear.

Our study had several limitations. First, our cohort was derived from a single institution with a relatively modest sample size, which may have constrained statistical power and limited the generalizability of our findings. Second, since our study focused on germline DNA from peripheral blood, somatic data from ovarian tissue (such as tumor-normal matched sequencing) was unavailable. Future studies integrating tumor-normal matched sequencing analyses may help further clarify the clonal origin of these alterations. Third, although we attempted to comprehensively analyze CHIP, the absolute number of identified CHIP carriers was limited due to our small sample size. However, our high mean sequencing depth of 112× ensured that CHIP clones with a VAF of 2% or higher were evaluated with high reliability and specificity. Fourth, since this study was conducted retrospectively, we were unable to capture longitudinal changes in CHIP VAF over time. Future large-scale prospective studies using deep sequencing may provide further insights into these clonal dynamics. Lastly, FH was ascertained solely through interviews with participants, which limited our ability to construct complete pedigrees and verify exact cancer diagnoses. Furthermore, our HC groups were skewed toward younger individuals, which may have introduced selection bias when compared to the HGSOC group. However, this study offers a unique strength in that we aimed to identify not only ovarian and breast cancers but also pancreatic and prostate cancers, aligning with the expanded hereditary breast and ovarian cancer syndrome definition and enhancing clinical relevance for comprehensive hereditary cancer risk assessment.

Taken together, our findings demonstrate genomic overlaps between ovarian cancer patients and family history-positive individuals, highlighting the importance of further in-depth functional studies. Although ovarian cancer, particularly high grade serous ovarian cancer, is characterized by remarkable intratumor heterogeneity [27] that necessitates extensive somatic variant research for therapeutic development, improving survival rates through early detection equally requires germline variant research. In addition, in-depth functional studies of P/LP germline variants could expand the repertoire of ovarian cancer susceptibility genes, ultimately enabling earlier diagnosis and improved survival. Given that universal germline screening in the general population remains impractical, the observed enrichment of P/LP germline variants among individuals with a family history of ovarian cancer is consistent with current clinical guidelines supporting multi-gene panel testing in this population.

## 4. Materials and Methods

### 4.1. The Study Population and WES Analyses of Blood-Derived DNA

This study included 231 participants recruited from Seoul St. Mary’s Hospital, Repubilic of Korea. The dataset comprised three groups: patients diagnosed with high-grade serous ovarian cancer (HGSOC, *n* = 39), healthy controls (HCs) with a FH of hereditary breast and ovarian cancer (HC-FH(+), *n* = 156), and HCs without a FH of these cancers (HC-FH(−), *n* = 36) (Table 1). This study was approved by the Institutional Review Board of Seoul St. Mary’s Hospital (Approval Nos.: KC17TESI0690 (25 October 2017), KC21TISI1016 (18 January 2022)). Patients were informed of the study and signed an informed consent sheet. All patient data were de-identified to maintain confidentiality. This research was conducted in accordance with the Declaration of Helsinki and all applicable ethical guidelines and regulations.

Genomic DNA was extracted from the buffy coat of peripheral blood using an Agilent SureSelect Human All Exon V8 probe set (Agilent Technologies, Santa Clara, CA, USA). WES was performed using 101 bp paired-end reads on an Illumina NovaSeq X sequencing platform (Illumina, San Diego, CA, USA). Further details of the WES procedure and supporting references are provided in the Supplementary Methods [28,29,30,31,32,33,34,35].

### 4.2. Germline Variant Calling and Filtering

Germline variants were called using GATK4 (Genome Analysis Toolkit, Version 4.3.0.0) Haplotypecaller and filtered using GATK4 SelectVariants and VariantFiltration based on GATK’s best practices for short germline variants. Functional annotation was performed using ANNOVAR (Annotate Variation, Version 8 June 2020) [36]. The germline analysis workflow is shown (Figure 1). In total, 3691 genes from cancer-predisposing gene panels (Table S7) were selected to investigate high-confidence germline variants. For downstream analysis, variant pathogenicity was classified using ClinVar [37], and only P/LP variants were retained.

### 4.3. Data Analysis of P/LP Germline Mutations

To identify genetic risk variants associated with cancer in HGSOC, HC-FH(+), and HC-FH(−) groups, enrichment and comparative analyses were independently applied to groups using P/LP germline variants. Specifically, enrichment analysis was performed to characterize functional profiles in mutated genes in groups, whereas comparative analysis was conducted to determine whether variant frequencies in our cohorts were significantly increased when compared to normal control, public databases. Kyoto Encyclopedia of Genes and Genomes (KEGG) pathway enrichment analysis was conducted using the clusterProfiler R package (Version 4.10.1) [38,39]. A comparative analysis was performed by comparing P/LP germline variant frequencies in each group with those in East Asian (EAS) individuals from the Genome Aggregation Database (gnomAD, Version 4.1) cohort [40]. The gnomAD (EAS) cohort comprises 22,448 individuals of EAS ancestry. Pathways and variants with a Benjamini–Hochberg adjusted *p*-value ≤ 0.05 were considered statistically significant.

### 4.4. CHIP Variant Calling

In tumor-only mode, somatic variants in blood cells were identified using GATK4 MuTect2. A public panel of normals (https://storage.googleapis.com/gatk-best-practices/somatic-hg38/1000g_pon.hg38.vcf.gz, accessed on 25 December 2024) was used to eliminate frequent sequencing artifacts. Somatic variants were further examined using GATK4 FilterMutectCalls and functionally annotated using ANNOVAR. The criteria for somatic mutations in CHIP analysis are described in Figure 1 and the Supplementary Methods (available online). CHIP-related somatic mutations were limited to those within 74 genes known to drive clonal hematopoiesis and myeloid malignancies, which have been previously used to identify CHIP [19,41,42,43].

### 4.5. Data Analysis of P/LP Germline Mutations and CHIP

To reduce baseline imbalance between HGSOC and HC groups, 1:4 propensity score matching (PSM) was performed using MatchIt (Version 4.7.2) [44]. A multivariate logistic regression model was constructed to determine an association between cancer risk and variables including germline variants and CHIP. For germline variants, P/LP germline variants in ovarian cancer-related genes were selected based on NCCN guidelines for ovarian cancer [4]. Covariates included in PSM and the multivariate logistic model were age and FH.

### 4.6. Statistical Analysis

Two-sided Fisher’s exact tests were used to compare differences between two groups. When a feature was present in only one group, 0.5 was added to all cells for Fisher’s exact test calculations. Continuous data normality was assessed using Shapiro–Wilk tests. Wilcoxon rank-sum tests were used to evaluate differences between two groups with respect to age and the number of variants. All statistical analyses were performed in R software (Version 4.2.3). For multiple testing corrections, *p*-values were adjusted using the Benjamini–Hochberg method, with a significance threshold of ≤0.05 [45].

## 5. Conclusions

P/LP germline variants are important hereditary risk factors of ovarian cancer. Given that FH remains a robust clinical predictor, our study suggests that germline screening yields significant clinical utility for healthy individuals with a FH of HBOC-related cancers. Although the clinical significance remains unclear, the observed CHIP-related findings could be regarded as exploratory and hypothesis-generating. Our findings underscore the importance of genetic counseling, screening, and risk assessment for ovarian cancer in order to develop personalized prevention strategies. 

## Figures and Tables

**Table 1:** Participants’ clinical information.

Characteristics	HGSOC (*n* = 39)	HC-FH(+) (*n* = 156)	HC-FH(−) (*n* = 36)
Age (mean ± S.D.)	57.41 ± 11.65	39.9 ± 11.51	51.17 ± 9.71
20–29 years	-	29 (18.6%)	1 (2.8%)
30–39 years	1 (2.6%)	63 (40.4%)	2 (5.6%)
40–49 years	10 (25.6%)	33 (21.2%)	13 (36.1%)
50–59 years	10 (25.6%)	20 (12.8%)	14 (38.9%)
60–69 years	11 (28.2%)	9 (5.8%)	5 (13.9%)
70–79 years	5 (12.8%)	2 (1.3%)	1 (2.8%)
80–89 years	2 (5.1%)	-	-
Underlying disease (Hypertension, diabetes, thyroid disease, rheumatic disease, etc.)	12(30.7%)	43(27.5%)	5(13.9%)
Concurrent history of any cancer	3 (7.7%)	-	-
FH in third-degree relatives			
Histology			
HBOC-related cancers	3 (7.7%)	156 (100%)	-
Breast cancer (BC)	-	110 (70.5%)	-
Ovarian cancer (OC)	2 (5.1%)	63 (40.4%)	-
Prostate cancer (PC)	-	2 (1.3%)	-
Pancreatic cancer (PA)	1 (2.6%)	6 (3.8%)	-
Other cancers	11 (28.2%)	-	-
Unspecified cancers	2 (5.1%)	-	-
Number of HBOC-related FH	0.08 (0–1)	1.63 (1–5)	-
0	36 (92.3%)	-	36 (100%)
1	3 (7.7%)	79 (50.6%)	-
2	-	59 (37.8%)	-
3	-	15 (9.6%)	-
4	-	2 (1.3%)	-
5	-	1 (0.6%)	-

A dash (“-”) indicates that no sample was reported in this group. Abbreviation: HGSOC, High-grade serous ovarian cancer; HC, Healthy control; FH, Family history; SD, Standard deviation; HBOC, Hereditary breast and ovarian cancer.

## Data Availability

The data generated in this study are available in the Korea Sequence Read Archive in Korea Bioinformation Center and Korea Research Institute of Bioscience and Biotechnology (accession code: KAP240757) that are publicly accessible at https://kbds.re.kr/KRA.

## Supplementary Materials

The following supporting information can be downloaded at: https://www.mdpi.com/article/10.3390/ijms27125564/s1.

## Abbreviations

The following abbreviations are used in this manuscript:

ACMG	American College of Medical Genetics and Genomics
COSMIC	Catalogue of Somatic Mutations in Cancer
CHIP	Clonal hematopoiesis of indeterminate potential
B/LB	Benign or likely benign
CI	Confidence interval
EAS	East Asian
gnomAD	Genome Aggregation Database
HBOC	Hereditary breast and ovarian cancer
HC	Healthy controls
HC-FH(+)	Healthy controls with family history of HBOC-associated cancers
HC-FH(−)	Healthy controls without family history of HBOC-associated cancers
HGSOC	High-grade serous ovarian cancer
IRB	Institutional Review Board
KEGG	Kyoto Encyclopedia of Genes and Genomes
NCG	Network of Cancer Genes
OR	Odds ratio
P/LP	Pathogenic or likely pathogenic
SD	Standard deviation
VAF	Variant allele frequency
WES	Whole-exome sequencing
