# Supplementary material for: Comprehensive Germline Profiling of High-Grade Serous Ovarian Cancer Using Whole-Exome Sequencing
Source: Int J Mol Sci. 2026 Jun 19;27(12):5564. doi: 10.3390/ijms27125564 (PMC13299865; doi:10.3390/ijms27125564)
Supplement: Supplementary file 1 [file ijms-27-05564-s001.zip › Supplementary Methods_FF.pdf]

Supplementary Methods

DNA Extraction and Whole-Exome Sequencing (WES)

Genomic DNA was extracted from the buffy coat of peripheral blood obtained from 39 patients with HGSOC and 192 HCs. Exome capture libraries were prepared using the Agilent SureSelect Target Enrichment protocol (Agilent Technologies, Santa Clara, CA, USA) for the Illumina paired-end sequencing library (Version C2, December 2018), following manufacturer's instructions. In all samples, the SureSelect Human All Exon V8 probe set (Agilent Technologies, Santa Clara, CA, USA) was used. WES was performed using 101 bp paired-end reads on an Illumina NovaSeq X sequencing platform (Illumina, San Diego, CA, USA).

WES Data Processing

Low quality raw sequence reads and adapter sequences were trimmed using Trimmomatic (Version 0.39), followed by sequence quality assessment with FastQC (Version 0.11.9) [1,2]. Trimmed reads were aligned to the human reference genome (*hg38*) using Burrows-Wheeler Aligner-Maximal Exact Match (BWA-MEM, Version 0.7.17) [3]. The Genome Analysis Toolkit (GATK, Version 4.3.0.0) Best Practices workflow was used for coordinate sorting, duplicate read marking, and base quality score recalibration [4]. Aligned reads with a mapping quality < 20 were removed using SAMtools (Version 1.6) [5].

Germline Variant Calling and Filtering

High-confidence germline variants were identified based on the following thresholds: 1) Located in coding regions; 2) Non-synonymous or loss-of-function variants; 3) Genotype quality ≥ 20 and coverage depth ≥ 20; 4) VAF ≥ 25%; and 5) Minor allele frequency (MAF) ≤ 1% in Asian populations from the Genome Aggregation Database (gnomAD, Version 4.1) [6] and the 1000 Genomes Project (1KGP) [7], as well as in the Korean population from the Korean Variant Archive (KOVA) [8] and the Korean National Standard Reference Variome (KoVariome) [9]. To focus specifically on oncology-relevant genetic variants, these high-confidence germline variants were further restricted to 3,691 genes derived from established cancer-predisposing panels. This integrated gene set was designed to systematically investigate hereditary risk and included genes from the American College of Medical Genetics and Genomics (ACMG) Secondary Findings gene list (Version 3.2) [10], the BROCA Cancer Risk Panel (Version 14) [11], the Catalog of Somatic Mutations in the Cancer Gene Census (COSMIC, Version 99) [12], the Network of Cancer Genes (NCG, Version 7.1) [13], and other curated genes [14,15] (Supplementary Table S1). According to ClinVar, the pathogenicity of identified variants was classified as benign (B), likely benign (LB), variant of uncertain significance (VUS), likely pathogenic (LP), or pathogenic (P) [16]. For downstream analysis, only pathogenic or likely pathogenic (P/LP) variants were retained.

CHIP Variant Calling

To identify CHIP variants, a WES data analysis framework was established through a systematic literature review of previous large-scale NGS-based CHIP studies [17-20]. This consensus-driven framework was coupled with high mean sequencing coverage of 112x to robustly evaluate candidate mutations with a VAF greater than 2% and bioinformatically minimize the risk of potential technical false positives. Based on prior empirical down-sampling benchmarks [19], a ~100× sequencing depth is statistically essential for reliably capturing minor CHIP variants in the lower 5–10% VAF range, meaning that our 112× coverage offers adequate technical sensitivity for robust clonal evaluation. Putative somatic mutations were retained based on the following criteria: 1) Located in coding or splicing regions; 2) Non-synonymous or loss-of-function variants; 3) Coverage depth ≥ 20; 4) 2% ≤ VAF ≤ 35%; 5) MAF ≤ 1% in Asian populations from gnomAD [6] and 1KGP [7], and in the Korean population from KOVA [8] and KoVariome; [9] 6) alternate allele depth ≥ 3; 7) variants supported by at least one read from each strand; and 8) variants annotated as "PASS" or "weak_evidence" by GATK4 FilterMutectCall. Among prioritized variants, CHIP-related somatic mutations were limited to those within 74 genes known to drive clonal hematopoiesis and myeloid malignancies, which have been commonly used in previous studies to identify CHIP [17-20]. Subsequently, all putative somatic mutations were evaluated to determine whether the VAF showed a significant deviation from the expected germline allele distribution. Briefly, a one-sided binomial model with a success probability of 0.5 was applied and the number of tries calculated as the sum of reference and alternate allele read counts. The number of successes was defined as the read count of the alternate allele. Only variants with a Benjamini–Hochberg adjusted p-value < 0.05 in binomial tests were considered somatic CHIP mutations and selected for CHIP analysis.

References

1. Andrews, S. FastQC: a quality control tool for high throughput sequence data 2010. Available online: https://www.bioinformatics.babraham.ac.uk/projects/fastqc/ (accessed on 5 February 2025).
2. Bolger, A.M.; Lohse, M.; Usadel, B. Trimmomatic: a flexible trimmer for Illumina sequence data. *Bioinformatics* **2014,** 30, 2114–2120. https://doi.org/10.1093/bioinformatics/btu170.
3. Li, H.; Durbin, R. Fast and accurate short read alignment with Burrows-Wheeler transform. *Bioinformatics* **2009,** 25, 1754–1760. https://doi.org/10.1093/bioinformatics/btp324.
4. DePristo, M.A.; Banks, E.; Poplin, R.; Garimella, K.V.; Maguire, J.R.; Hartl, C.; Philippakis, A.A.; del Angel, G.; Rivas, M.A.; Hanna, M.; et al. A framework for variation discovery and genotyping using next-generation DNA sequencing data. *Nat. Genet.* **2011,** 43, 491–498. https://doi.org/10.1038/ng.806.
5. Li, H.; Handsaker, B.; Wysoker, A.; Fennell, T.; Ruan, J.; Homer, N.; Marth, G.; Abecasis, G.; Durbin, R.; 1000 Genome Project Data Processing Subgroup. The Sequence Alignment/Map format and SAMtools. *Bioinformatics* **2009,** 25, 2078–2079. https://doi.org/10.1093/bioinformatics/btp352.
6. Karczewski, K. J.; Francioli, L. C.; Tiao, G.; Cummings, B. B.; Alfoldi, J.; Wang, Q.; Collins, R. L.; Laricchia, K. M.; Ganna, A.; Birnbaum, D. P.; et al. The mutational constraint spectrum quantified from variation in 141,456 humans. *Nature* **2020,** 581, 434–443. https://doi.org/10.1038/s41586-020-2308-7.
7. 7. 1000 Genomes Project Consortium; Auton, A.; Brooks, L.D.; Durbin, R.M.; Garrison, E.P.; Kang, H.M.; Korbel, J.O.; Marchini, J.L.; McCarthy, S.; McVean, G.A.; et al. A global reference for human genetic variation. *Nature* **2015,** 526, 68–74. https://doi.org/10.1038/nature15393.
8. Lee, S.; Seo, J.; Park, J.; Nam, J.Y.; Choi, A.; Ignatius, J.S.; Bjornson, R.D.; Chae, J.H.; Jang, I.J.; Lee, S.; et al. Korean Variant Archive (KOVA): a reference database of genetic variations in the Korean population. *Sci. Rep.* **2017,** 7, 4287. https://doi.org/10.1038/s41598-017-04642-4.
9. Kim, J.; Weber, J.A.; Jho, S.; Jang, J.; Jun, J.; Cho, Y.S.; Kim, H.M.; Kim, H.; Kim, Y.; Chung, O.; et al. KoVariome: Korean National Standard Reference Variome database of whole genomes with comprehensive SNV, indel, CNV, and SV analyses. *Sci. Rep.* **2018,** 8, 5677. https://doi.org/10.1038/s41598-018-23837-x.
10. Miller, D. T.; Lee, K.; Abul-Husn, N. S.; Amendola, L. M.; Brothers, K.; Chung, W. K.; Gollob, M. H.; Gordon, A. S.; Harrison, S. M.; Hershberger, R. E.; et al. ACMG SF v3.2 list for reporting of secondary findings in clinical exome and genome sequencing: A policy statement of the American College of Medical Genetics and Genomics (ACMG). *Genet. Med.* **2023,** 25, 100866. https://doi.org/10.1016/j.gim.2023.100866.
11. Shirts, B. H.; Casadei, S.; Jacobson, A. L.; Lee, M. K.; Gulsuner, S.; Bennett, R. L.; Miller, M.; Hall, S. A.; Hampel, H.; Hisama, F. M.; et al. Improving performance of multigene panels for genomic analysis of cancer predisposition. *Genet. Med.* **2016,** 18, 974–981. https://doi.org/10.1038/gim.2015.212.
12. Tate, J. G.; Bamford, S.; Jubb, H. C.; Sondka, Z.; Beare, D. M.; Bindal, N.; Boutselakis, H.; Cole, C. G.; Creatore, C.; Dawson, E.; et al. COSMIC: the Catalogue of Somatic Mutations in Cancer. *Nucleic Acids Res.* **2019,** 47, D941–D947. https://doi.org/10.1093/nar/gky1015.
13. Repana, D.; Nulsen, J.; Dressler, L.; Bortolomeazzi, M.; Venkata, S. K.; Tourna, A.; Yakovleva, A.; Palmieri, T.; Ciccarelli, F. D. The Network of Cancer Genes (NCG): a comprehensive catalogue of known and candidate cancer genes from cancer sequencing screens. *Genome Biol.* **2019,** 20, 1. https://doi.org/10.1186/s13059-018-1612-0.
14. Rahman, N. Realizing the promise of cancer predisposition genes. *Nature* **2014,** 505, 302–308. https://doi.org/10.1038/nature12981.
15. Knijnenburg, T. A.; Wang, L.; Zimmermann, M. T.; Chambwe, N.; Gao, G. F.; Cherniack, A. D.; Fan, H.; Shen, H.; Way, G. P.; Greene, C. S.; et al. Genomic and Molecular Landscape of DNA Damage Repair

Deficiency across The Cancer Genome Atlas. *Cell Rep.* **2018,** 23, 239–254.e6. https://doi.org/10.1016/j.celrep.2018.03.076.

16. Landrum, M. J.; Lee, J. M.; Riley, G. R.; Jang, W.; Rubinstein, W. S.; Church, D. M.; Maglott, D. R. ClinVar: public archive of relationships among sequence variation and human phenotype. *Nucleic Acids Res.* **2014,** 42, D980–985. https://doi.org/10.1093/nar/gkt1113.
17. Wang, A.; Xu, Y.; Yu, Y.; Nead, K. T.; Kim, T.; Xu, K.; Dadaev, T.; Saunders, E.; Sheng, X.; Wan, P.; et al. Clonal hematopoiesis and risk of prostate cancer in large samples of European ancestry men. *Hum. Mol. Genet.* **2023,** 32, 489–495. https://doi.org/10.1093/hmg/ddac214.
18. Jiang, Z.; Li, Y.; Yan, C.; Zhang, X.; Zhang, Q.; Li, J.; Tian, X.; Qiu, M.; Liang, Z.; Ma, S.; et al. Clonal hematopoiesis of indeterminate potential in patients with acute coronary syndrome undergoing percutaneous coronary intervention in the absence of traditional risk factors. *Clin. Res. Cardiol.* **2023,** 112, 506–517. https://doi.org/10.1007/s00392-022-02039-6.
19. Bick, A. G.; Weinstock, J. S.; Nandakumar, S. K.; Fulco, C. P.; Bao, E. L.; Zekavat, S. M.; Szeto, M. D.; Liao, X.; Leventhal, M. J.; Nasser, J.; et al. Inherited causes of clonal haematopoiesis in 97,691 whole genomes. *Nature* **2020,** 586, 763–768. https://doi.org/10.1038/s41586-020-2819-2.
20. Yu, Z.; Fidler, T. P.; Ruan, Y.; Vlasschaert, C.; Nakao, T.; Uddin, M. M.; Mack, T.; Niroula, A.; Heimlich, J. B.; Zekavat, S. M.; et al. Genetic modification of inflammation- and clonal hematopoiesis-associated cardiovascular risk. *J. Clin. Invest.* **2023,** 133, e168597. https://doi.org/10.1172/JCI168597.
