# Supplementary figures and images for: Comprehensive Germline Profiling of High-Grade Serous Ovarian Cancer Using Whole-Exome Sequencing
Source: Int J Mol Sci. 2026 Jun 19;27(12):5564. doi: 10.3390/ijms27125564 (PMC13299865; doi:10.3390/ijms27125564)

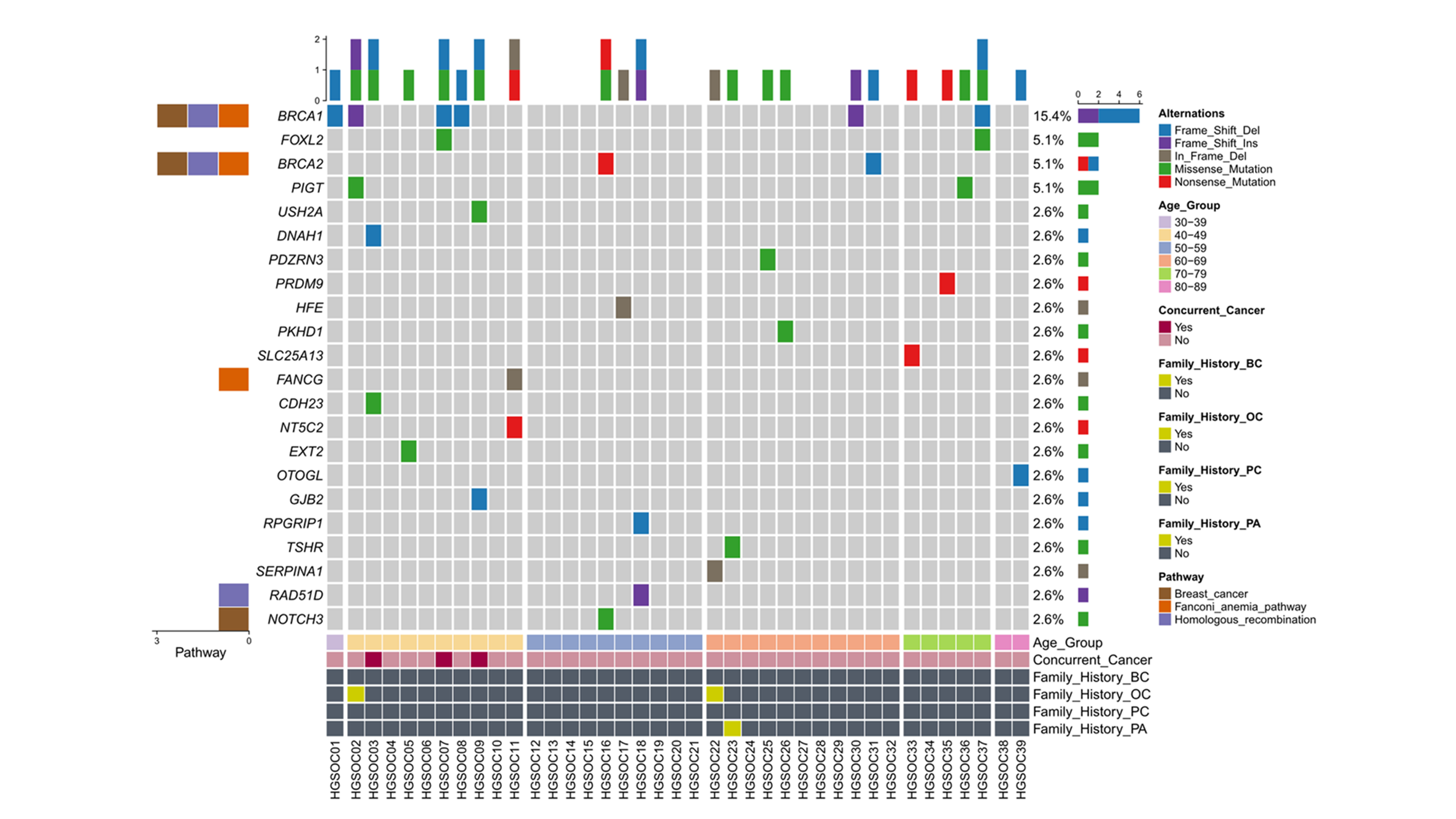

Supplement: Supplementary file 1 [file ijms-27-05564-s001.zip › Figure S1_300dpi;20cm(w).tif]

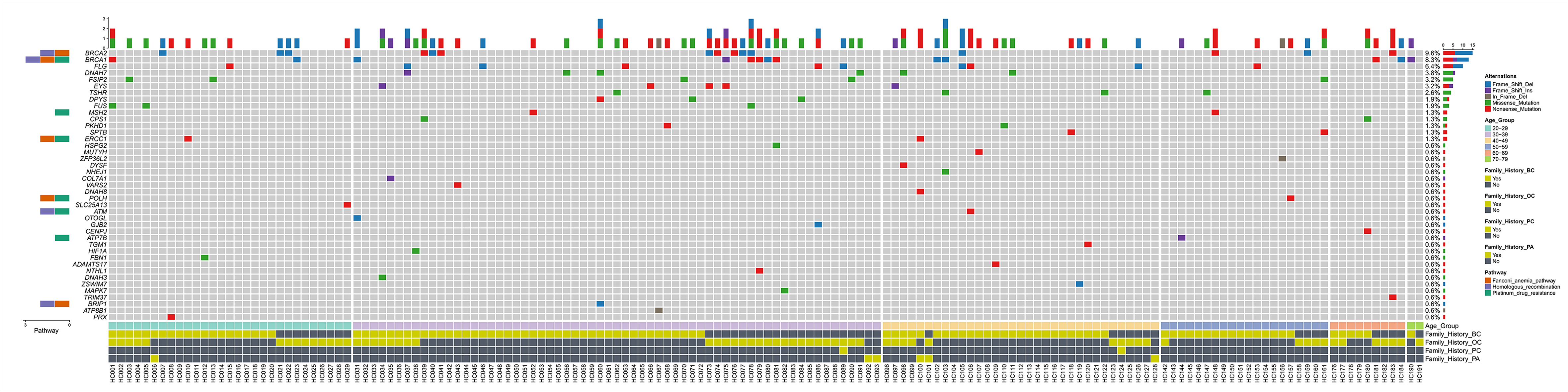

Supplement: Supplementary file 1 [file ijms-27-05564-s001.zip › Figure S2_300dpi;40cm(w).tif]

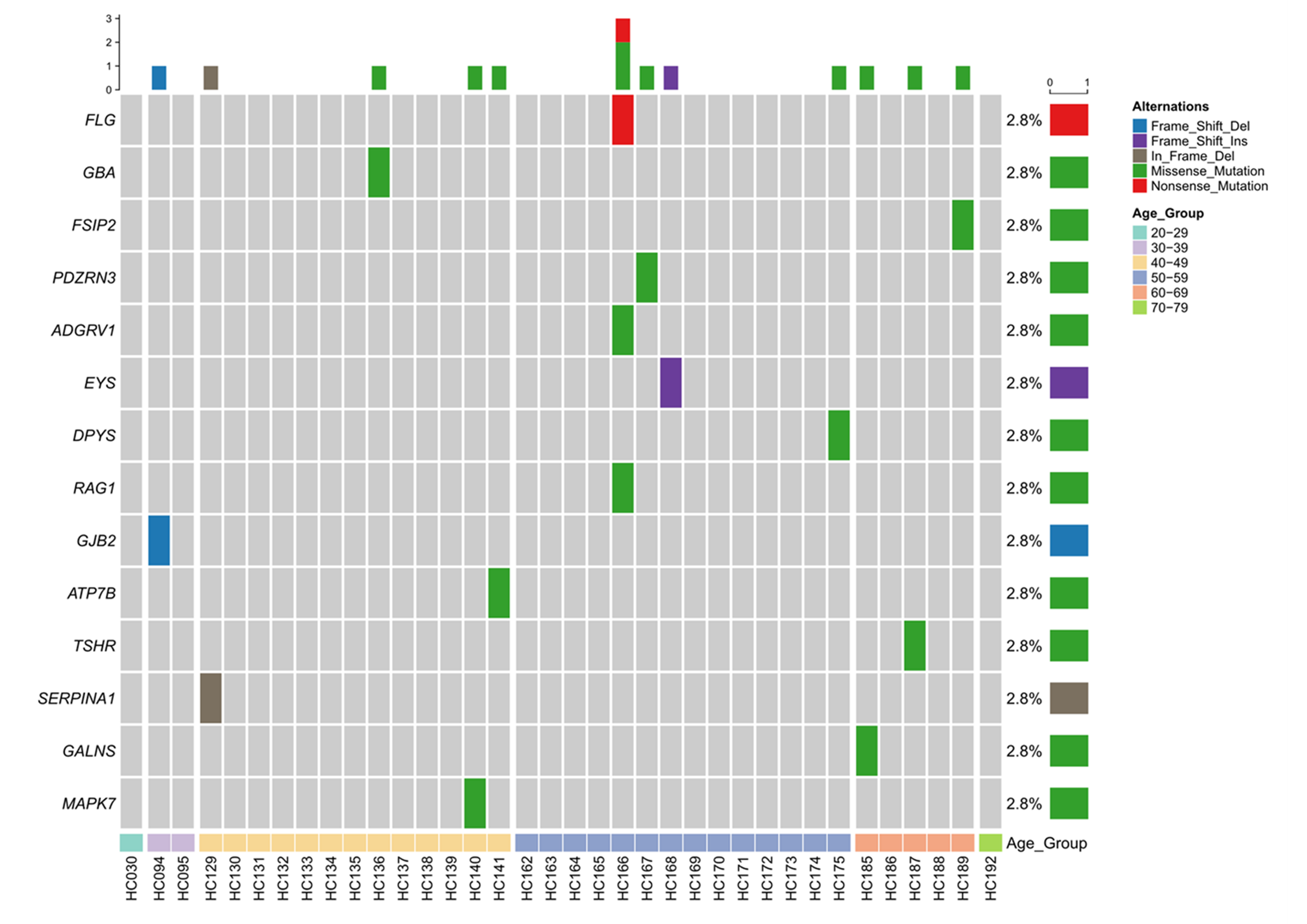

Supplement: Supplementary file 1 [file ijms-27-05564-s001.zip › Figure S3_300dpi;20cm(w).tif]

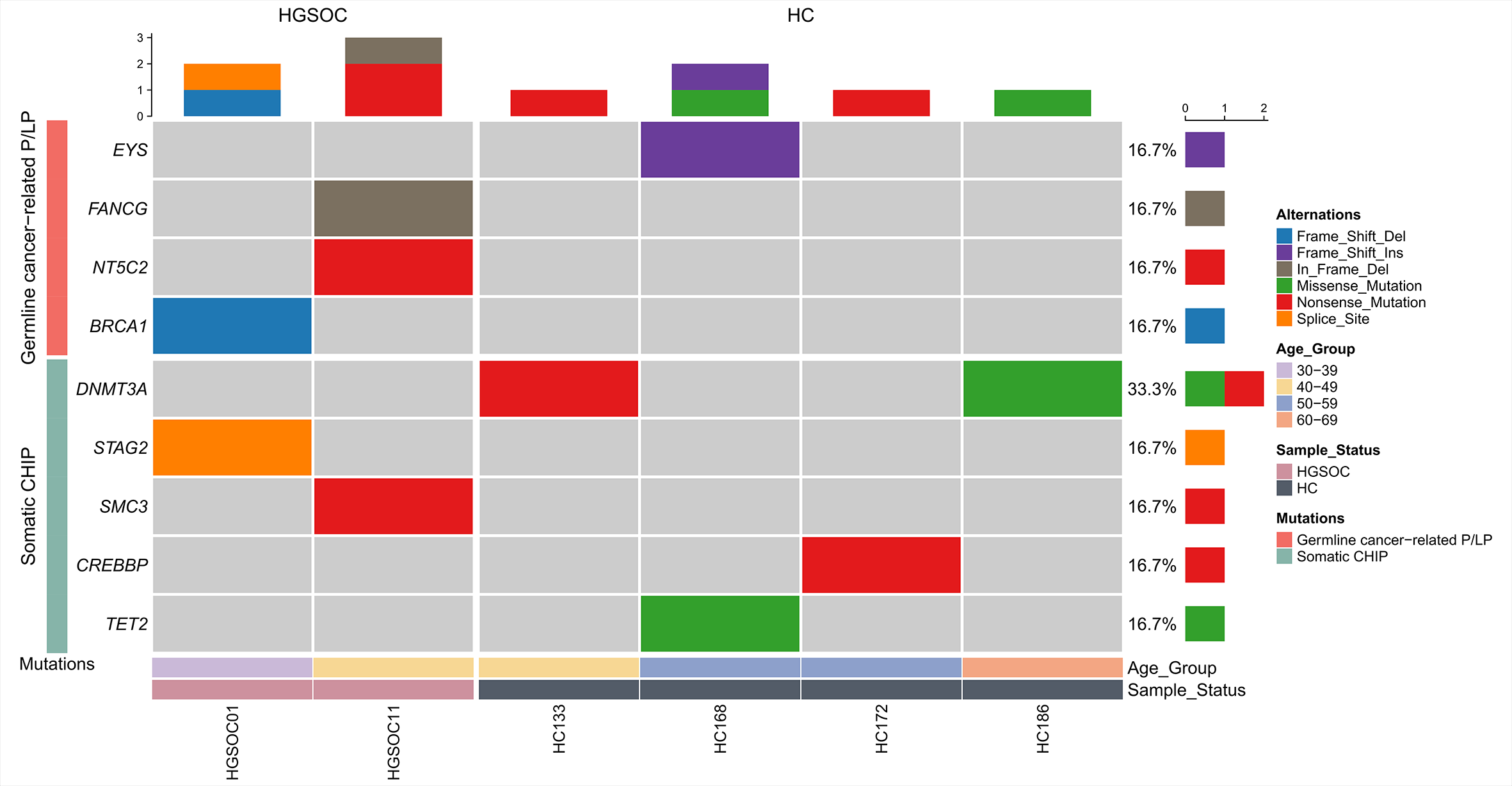

Supplement: Supplementary file 1 [file ijms-27-05564-s001.zip › Figure S4_300dpi;20cm(w).tif]
